# The impact of acute coronary syndrome on long-term survival in cancer patients

**DOI:** 10.1186/s40959-026-00451-9

**Published:** 2026-02-07

**Authors:** Daniel Finke, Markus B. Heckmann, Jessica M. Schug, Lukas F. Entenmann, Hauke Hund, Hugo A. Katus, Norbert Frey, Lorenz H. Lehmann

**Affiliations:** 1 Department of Cardiology, Heidelberg University Hospital, Heidelberg, Germany; 2 German Centre for Cardiovascular Research (DZHK), Heidelberg/Mannheim, Germany; 3 Deutsches Krebsforschungszentrum, Heidelberg, Germany

**Keywords:** Cardio-Oncology, Acute coronary syndrome, Cardiovascular risk stratification, PCI, Cancer patient care

## Abstract

**Background:**

Although cancer and atherosclerosis are based on shared risk factors, the impact of the acute coronary syndrome (ACS) on long-term survival in cancer patients is still unclear.

**Methods:**

We retrospectively analyzed 441 patients with initial diagnosis of cancer prior to ACS who were subjected to cardiac catheterization (CC) with a follow-up of at median 4.78 years (IQR: 0.58–9.33). These patients were compared to non-cancer ACS patients via propensity score matching according to the cardiac risk factors age, sex, arterial hypertension and diabetes.

**Results:**

We found ACS, and overall Non-ST-elevation myocardial infarction (NSTEMI), as a relevant predictor of outcome in cancer patients with the highest mortality rates (88 deaths (40%) in non-cancer patients with NSTEMI vs. 174 deaths (64.9%), in the respective cancer patients *p* < 0.001). In multivariable COX models including other comorbidities, cancer was found to independently influence mortality as well as cardiovascular hospital readmission (HR all-cause mortality: 1.47, *p* < 0.001, HR readmission: 1.54, *p* < 0.001). Other risk factors, such as renal dysfunction, diabetes or impaired left ventricular function showed comparable influence on mortality. Percutaneous coronary intervention (PCI) rates did not differ between the groups (228/441 non-cancer patients (51.7%) vs. 227/441 cancer patients (51.5%), *p* = 0.95), but optimized medical treatment was less present in the cancer group.

In a subgroup of cancer patients with available biomarker data, NT-proBNP, but not hs-cTnT, was found to be useful for risk stratification (HR univariate: 1.28, HR multivariate considering risk factors and hs-cTnT: 1.26, *p* < 0.001 .

**Conclusions:**

Our data indicate a relevant effect of ACS, especially NSTEMI, in cancer patients with impact on long-term mortality and cardiac morbidity. Cancer patients with NSTEMI warrant special care, considering comorbidities and optimized medical treatment of the cardiac disease.

**Supplementary Information:**

The online version contains supplementary material available at 10.1186/s40959-026-00451-9.

## Introduction

Cancer patients are at increased risk of coronary artery disease (CAD) and acute coronary syndromes (ACS) due to their malignant disease, the related therapies and shared risk factors for cancer and atherosclerosis [1–3]. Advances in cancer therapies with the use of potentially cardiotoxic agents, lately e.g., immune checkpoint inhibitors or tyrosine kinase inhibitors, achieved major improvements in cancer survival [4, 5]. Given improved outcomes, cardiovascular complications of the underlying disease are becoming increasingly important [6]. In cancer types with many long-term survivors, such as breast cancer, cardiovascular disease is a leading cause of mortality [7]. 

In the 2022 ESC cardiooncology guidelines and current publications in the field, there is an emphasis on the cardiological risk assessment of oncological patients using e.g., cardiac biomarkers and cardiac imaging, mainly in stable patients, not suffering from an acute cardiac event [8–12]. 

Generally, there are clear guidelines on the management of ACS [13]. Nevertheless, clinical practice shows uncertainties in the implementation of these established guidelines in patients with cancer. Reasons are mostly attributed to difficulties in anti-platelet treatments after percutaneous coronary interventions (PCI) and bleeding complications [14, 15]. Given the reduced life expectancy associated with a cancer diagnosis, the clinical value of invasive cardiac assessments remains a subject of debate [15, 16]. 

Still, detailed data are lacking on both the long-term survival of patients with acute coronary syndrome (ACS) and concomitant cancer, as well as on effective strategies for their risk stratification. There is a gap in knowledge, if biomarkers can be used for risk stratification in cancer patients when suffering from ACS. Further it is not known, if ACS relevantly alters the morbidity of cancer patients.

Given the high overlap of cancer and risk factors for ACS, we aimed to assess, if the co-occurrence of cancer and ACS is relevant for the patients’ survival. The study also aims to evaluate the clinical features such as ACS classification (unstable Angina pectoris (uAP), ST- or Non-ST Elevation myocardial infarction (STEMI/NSTEMI)), other co-morbidities and cardiac assessments (left ventricular ejection fraction (LVEF) and CAD) in order to look for outcome-relevant potential risk factors in this patient collective. Lastly, we assessed re-hospitalization due to cardiac disease as an indicator for morbidity that is not only cancer-dependent.

These insights may contribute to improvements in clinical care for cancer patients that are suffering from ACS.

## Materials and methods

### Study design and patients

This retrospective matched study was conducted at the University Hospital Heidelberg. Data were acquired from patients who underwent cardiac catheterization (CC) at University Hospital Heidelberg between January 1, 2006 and December 31, 2017. The first CC of each patient was considered for the analysis. We selected a subgroup of patients who were subjected to CC in an emergency setting. These patients’ medical records were screened for ACS subtype specification (ST-elevation myocardial infarction (STEMI), NSTEMI and unstable Angina (uAP)). The cancer group consists of patients with an initial diagnosis of the malignant disease before CC. We included patients with the diagnosis of gastrointestinal cancer (ICD-10: C15, C16, C18, C19, C20, C22, C24, C25, C78.7, D01.0), lung cancer (ICD-10: C34, C78.0), breast cancer (ICD-10: C50, D05), melanoma/skin tumors (ICD-10: C43, C44, D03, D04), prostate cancer (ICD-10: C61), leukemia (ICD-10: C91, C92, C93, D45, D46, D47), lymphoma (ICD-10: C81, C82, C83, C84, C85), kidney tumors (ICD-10: C64, C65), soft tissue tumors (ICD-10: C46, C48, C49) and others (ICD-10: C02, C03, C07, C09, C11, C14, C32, C37, C40, C51, C53, C54, C55, C56, C60, C62, C67, C69, C71, C73, C80, C90, D00.0, D09, D32, D33, D35, D39, D41, D43, Y35.7). Non-cancer patients were matched according to age, sex, diabetes and arterial hypertension to serve as a control group.

The study protocol was approved by the ethics committee of the Medical Faculty of the University of Heidelberg (S-286/2017, 390/2011). The investigation conformed with the principles outlined in the *Declaration of Helsinki.*

### Data acquisition

Patient specific data were obtained in the clinical routine and were extracted from electronic medical records including demographic data, ACS classifications, laboratory results (including creatinine, hemoglobin (Hb), C-reactive protein (CRP), leucocyte count, high-sensitivity cardiac troponin T (hs-cTnT) and N-terminal prohormone of brain natriuretic peptide (NT-proBNP)), angiographic measurements and hospital readmission at University Hospital Heidelberg on a cardiological ward using the cardiac Research Data Warehouse (RWH). LVEF was assessed during CC via laevo-cardiography. Laboratory values that were assessed between seven days prior to seven days after the CC were considered for further analysis. Generally, the values closest to CC were taken into account. In terms of hs-cTnT, that is often measured repeatedly for ACS rule-out, we took the highest value. The oncological diagnosis (ICD-10 code), the metastasis status, date of diagnosis and type of treatment were retrieved from the Clinical Registry of the National Centre for Tumor Diseases (NCT) Heidelberg. Data of all-cause mortality and date of death were obtained from the registration office from the patients’ place of residence. Follow-up of ACM was analyzed by October 1, 2020.

In the clinical routine, in a subgroup of patients hs-cTnt and NT-proBNP was assessed. Measurement of hs-cTnT in plasma samples was performed using the Elecsys^®^ Troponin T high sensitive hs-cTnT assay (Roche Diagnostics) in the central laboratory at Heidelberg University Hospital. Limit of blank (LoB), Limit of detection (LoD), 10% coefficient of variation (CV) and 99th percentile cut-off values for the hs-cTnT assay were 3 ng/L, 5 ng/L, 13 ng/L and 14 ng/L. N-terminal pro brain‐type natriuretic peptide was measured at the same timepoint using the Stratus^®^ CS Acute Care™ NT‐proBNP assay (Siemens AG, Berlin and Munich, Germany).

### Definitions

Impairments in the LVEF were defined as previously published and recommended in the guidelines of the European Society of Cardiology (ESC) from 2021. A reduced left ventricular ejection fraction (rEF) was defined as LVEF ≤ 40% [17]. Coronary stenosis ≥ 50% were used as cut-off for the occurrence CAD [18]. The diagnoses of COPD, stroke, diabetes and arterial hypertension prior to the CC were taken the medical reports. We used the cut-off Hb < 10 g/dl for detection of a relevant anemia with known impact on the outcome of heart failure patients as published earlier [19]. As a measure of kidney dysfunction we used the glomerular filtration rate (GFR) with a cutoff of 30 ml/min/1,73m^2^ for severely decreased kidney function and renal failure as suggested by the KDIGO [20]. An active oncological treatment was defined as chemotherapy or radiation that was administered within 6 months before the time of ACS.

### Statistical analysis

In order to compare continuous variables according to cancer status, we used the Wilcoxon-rank sum test. The variables were presented as median values with interquartile range (IQR). Dichotomic data were compared using the Chi-squared test and is presented as numbers of events and percentages. A confidence interval of 95% was considered significant.

1:1 propensity score matching was performed with the use of *MatchIt* package (version 4.5.4) according to age, sex, diabetes and arterial hypertension. Nearest neighbor matching was performed. The distance measure was estimated via logistic regression. No units were discarded before matching. Thus, we were able to obtain an adequate balance of propensity scores between the cancer and non-cancer group (Supplemental Fig. 1).

Multivariable COX models were calculated in R (version 4.3.1) using the *survival* package (version 3.7.0). The model was illustrated as a forest plot using the *survminer* package (version 0.4.9).

Survival analysis shown in Kaplan Meier curves were also calculated in R using the survival package (version 3.7.0) and illustrated with help of the *survminer* package (version 0.4.9). Adjusted survival curves were built with the *adjustedCurves* package (version 0.11.2). Survival in dependence of ACS classification was adjusted to diabetes, aHT, Age > 65, male sex, rEF, COPD and prior stroke using a Cox multivariable model (Supplemental Fig. 2). The time-to-event in survival analyses was defined as the difference between the date of the CC due to ACS and the date of death. Data were censored when the observation interval was lower than 5 years. The log-rank test was used to determine differences in survival. A *p*-value < 0.05 was considered significant.

To account for competing risks in the time-to-event analyses, we employed Fine and Gray’s subdistribution hazard model for competing risk regression. Specifically, we modeled the cumulative incidence of rehospitalization events, considering death as a competing event. Models were fitted, subdistribution hazard ratios (SHRs) and their corresponding 95% confidence intervals (CIs) were estimated using the *cmprsk* package (version 2.2.12), and results were organized with the *finalfit* package (version 1.0.8).

Tables were created with the use of packages *tableone* (version 0.13.2), *flextable* (version 0.9.2) and *utile.tables* (version 0.3.0). Data wrangling was supported by the *tidyverse* package (version 2.0.0). Figures were composed in Adobe Illustrator (version 29.0.1). Bar charts and pie charts were illustrated in GraphPad Prism (version 10.4.1.).

## Results

**Fig. 1:**
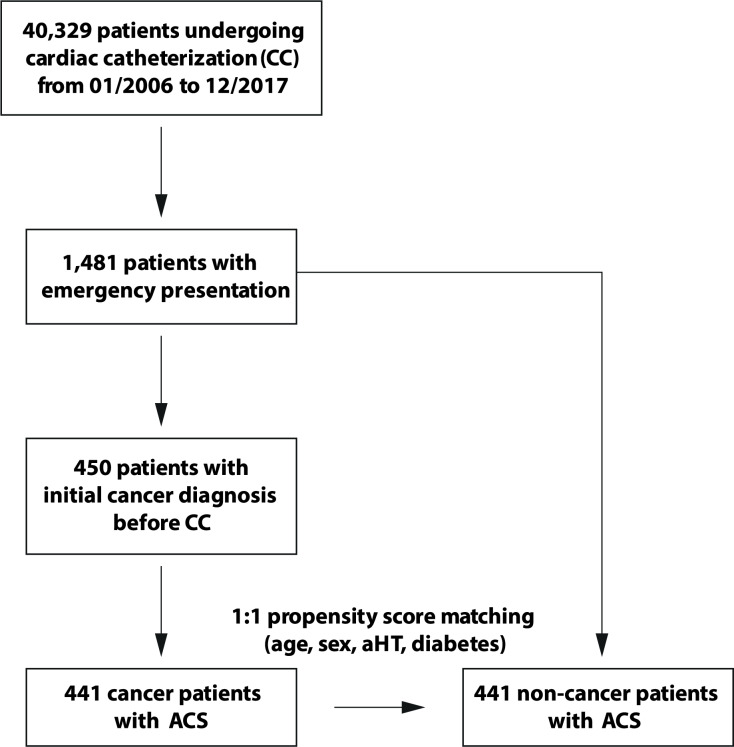
Flowchart of patient selection. 40,329 patients underwent cardiac catheterization. 1,481 of these patients presented as an emergency examination. Among those, we found 450 patients with a prior diagnosis of cancer. 441 cancer patients could be verified as ACS cases. Non-cancer controls with ACS were propensity score matched according to age, sex, arterial hypertension (aHT) and diabetes

40,329 patients underwent CC between January 2006 and December 2017 at University Hospital Heidelberg. Among those, 3,666 patients were diagnosed with cancer prior to CC. Risk stratification using cardiac parameters in the total cancer group including LVEF, CAD and biomarkers was published earlier [21]. As a subgroup, 1,481 patients with an emergency presentation were identified in the present analysis. 450 patients were diagnosed with cancer prior to the presentation for CC, with nine patients being excluded due to a missing diagnosis of ACS. 441 cancer patients and 441 propensity score matched non-cancer patients were followed up for further analysis (Fig. 1, Suppl. Figure 1).

The median age of all patients in the study was 80.1 years, 67.1% of patients were male, 69.6% were diagnosed with arterial hypertension and 28.3% were diabetic. These parameters were used for propensity score matching and, thus, equally distributed in the cancer and non-cancer group that were further analyzed. The most prevalent malignant diseases in the patients of this study were gastrointestinal cancer (15.6%), basal cell tumors (11.8%) and breast cancer (8.6%). Metastasis were known in 5% of cancer patients. At the time of ACS, 11.8% of patients had an active oncological therapy within the last 6 months. Cancer patients had significantly more diagnoses of comorbidities, such as COPD or prior stroke. Neither left ventricular function nor high-grade coronary stenosis, nor the cardiac biomarker hs-cTnT, nor NT-proBNP showed any relevant differences between the two groups (Suppl. Figure 2, Table 1). Cancer patients were more frequently diagnosed with NSTEMI (49.9% vs. 60.8%, *p* = 0.001) and less frequently with STEMI (41% vs. 29.7%, *p* < 0.001). PCI rates (51.7% vs. 51.5%, *p* = 0.945) and the frequency of coronary artery bypass graft (CABG) surgery (6.8% vs. 4.5%, *p* = 0.145) did not differ significantly between non-cancer and cancer patients. In NSTEMI patients, the rate of PCI was slightly lower than in the total cohort and comparable between the groups (Suppl. Figure 3). Medical therapies, including anti-platelet therapies, heart failure medication and statins were significantly less frequently administered in the cancer group (Table 1).

**Table 1:** Patient characteristics according to cancer diagnosis. ACE/AT1-inhibitors: angiotensin-converting enzyme and angiotensin II type 1 receptor inhibitors, ACS: acute coronary syndrome, CABG: coronary artery bypass graft, CAD: coronary artery disease, COPD: chronic obstructive pulmonary disease, hs-cTnT: high-sensitivity cardiac troponin T, LAD: left anterior descending artery, LCA: left coronary artery, LCX: left circumflex artery, LDH: lactate dehydrogenase, LVEF: left ventricular ejection fraction, mref: mildly reduced ejection fraction, NOAC: non-vitamin K antagonist anticoagulant, NSTEMI: Non-ST-elevation myocardial infarction, NT-proBNP: N-terminal prohormone of brain natriuretic peptide, PCI: percutaneous coronary intervention, pEF: preserved ejection fraction, RCA: right coronary artery, rEF: reduced ejection fraction, STEMI: ST-elevation myocardial infarction, uAP: unstable angina

Patients	Overall (*n* = 882)	Non-cancer (*n* = 441)	Cancer (*n* = 441)	*p*-value
Age [years] (mean ± SD)	80.1 ± 11.4	80.1 ± 11.5	80.2 ± 11.3	0.9433
Male sex (%)	592 (67.1%)	293 (66.4%)	299 (67.8%)	0.667
Medical history				
Arterial hypertension	614 (69.6%)	311 (70.5%)	303 (68.7%)	0.558
Diabetes	250 (28.3%)	124 (28.1%)	126 (28.6%)	0.881
Atrial fibrillation	128 (14.5%)	60 (13.6%)	68 (15.4%)	0.444
Heart failure	119 (13.5%)	52 (11.8%)	67 (15.2%)	0.139
COPD	88 (10%)	34 (7.7%)	54 (12.2%)	0.025
Stroke	75 (8.5%)	28 (6.3%)	47 (10.7%)	0.022
Cancer history				
Time difference cancer diagnosis to ACS [years] (mean ± SD)	6.8 ± 7.13		6.8 ± 7.13	
Melanoma	12 (1.4%)		12 (2.7%)	
Squamous cell tumor	21 (2.4%)		21 (4.8%)	
Basal cell tumor	52 (5.9%)		52 (11.8%)	
Other skin tumor	5 (0.6%)		5 (1.1%)	
Gastrointestinal tumor	69 (7.8%)		69 (15.6%)	
Prostate cancer	53 (6%)		53 (12%)	
Breast cancer	38 (4.3%)		38 (8.6%)	
Lung cancer	32 (3.6%)		32 (7.3%)	
Lymphoma	30 (3.4%)		30 (6.8%)	
Leukemia	24 (0.3%)		24 (5.4%)	
Kidney tumor	13 (1.5%)		13 (2.9%)	
Soft tissue tumor	12 (1.4%)		12 (2.7%)	
Other tumor	80 (9.1%)		80 (18.1%)	
Known metastatic disease	22 (2.5%)		22 (5%)	
Known oncologic therapy				
Active therapy	52 (5.9%)		52 (11.8%)	
Chemotherapy	73 (4.3%)		73 (16.6%)	
Radiotherapy	139 (15.8%)		139 (31.5%)	
Operation	102 (11.6%)		102 (23.1%)	
Stemcell transplantation	3 (0.3%)		3 (0.7%)	
Other therapy	30 (3.4%)		30 (6.8%)	
LVEF				
pEF	171 (19.4%)	89 (20.2%)	82 (18.6%)	0.551
mrEF	193 (21.9%)	108 (24.5%)	85 (19.3%)	0.061
rEF	421 (47.7%)	193 (43.8%)	228 (51.7%)	0.021
CAD (any stenosis ≥ 50%)	740 (83.9%)	374 (84.8%)	366 (83%)	0.464
Proximal coronary stenoses (≥ 90%)				
High-grade stenosis (LCA, main stem)	31 (3.5%)	16 (3.6%)	15 (3.4%)	0.855
High-grade stenosis (LAD)	77 (8.7%)	42 (9.5%)	35 (7.9%)	0.404
High-grade stenosis (LCX)	49 (5.6%)	27 (6.1%)	22 (5%)	0.462
High-grade stenosis (RCA)	102 (11.6%)	50 (11.3%)	52 (11.8%)	0.833
Lab. results (mean ± SD)				
Hb [mg/dl]	12.4 ± 2.3 (*n* = 860)	12.9 ± 2.1 ( *n* = 434)	11.8 ± 2.3 (*n* = 426)	< 0.0001
Leukocytes [number/nl]	11 ± 7 (*n* = 844)	11 ± 5 (*n* = 432)	12 ± 9 (*n* = 412)	0.579
Platelets [number/nl]	248 ± 110 (*n* = 858)	243 ± 84 (*n* = 434)	253 ± 131 (*n* = 424)	0.1606
GFR [ml/min]	66.3 ± 25,9 (*n* = 869)	68.7 ± 25.3 (*n* = 438)	63.9 ± 26.3 (*n* = 431)	0.0007
C-reactive protein [mg/dl]	55 ± 63.5 (*n* = 678)	46 ± 57.9 (*n* = 330)	64 ± 67.3 (*n* = 348)	0.0002
LDH [U/l]	269.2 ± 93.9 (*n* = 118)	255.1 ± 85.2 (*n* = 45)	278 ± 98.5 (*n* = 73)	0.1886
Hs-cTnT [ng/l]	2998.1 ± 7236.3 (*n* = 528)	3423.2 ± 8708 (*n* = 238)	2649 ± 5747.3 (*n* = 290)	0.24
NT-proBNP [ng/l]	7385 ± 14233.1 (*n* = 252)	6074 ± 10727.3 (*n* = 142)	9078 ± 17677.9 (*n* = 110)	0.1179
ACS and therapy				
NSTEMI	488 (55.3%)	220 (49.9%)	268 (60.8%)	0.001
STEMI	312 (35.4%)	181 (41%)	131 (29.7%)	< 0.001
uAP	82 (9.3%)	40 (9.1%)	42 (9.5%)	0.817
PCI	455 (51.6%)	228 (51.7%)	227 (51.5%)	0.946
CABG	50 (5.7%)	30 (6.8%)	20 (4.5%)	0.145
Medication				
Aspirin	694 (78.7%)	363 (82.3%)	331 (75.1%)	0.009
Clopidogrel	388 (44%)	212 (48.1%)	176 (39.9%)	0.015
Ticagrelor	158 (17.9%)	71 (16.1%)	87 (19.7%)	0.16
Prasugrel	26 (2.9%)	19 (4.3%)	7 (1.6%)	0.015
Heparin	188 (21.3%)	87 (19.7%)	101 (22.9%)	0.25
Marcumar	83 (9.4%)	46 (10.4%)	37 (8.4%)	0.299
NOAC	25 (2.8%)	9 (2%)	16 (3.6%)	0.156
Beta-receptor blocker	676 (76.6%)	355 (80.5%)	321 (72.8%)	0.007
Statins	659 (74.7%)	351 (79.6%)	308 (69.8%)	0.001
ACE/AT1-Inhibitors	643 (72.9%)	346 (78.5%)	297 (67.3%)	< 0.001
Aldosterone antagonist	134 (15.2%)	70 (15.9%)	64 (14.5%)	0.574
Outcome				
5 year all-cause mortality	391 (44,3%)	148 (33,6%)	243 (55,1%)	< 0.001
Cardiac Readmission [5 years]	569 (64.5%)	254 (57.6%)	315 (71.4%)	< 0.001
Median survival [months] (median ± SD)	13.5 ± 40.8	27 ± 46.9	8.5 ± 33.5	< 0.0001

### 5-year overall survival is dependent on ACS classification

**Fig. 2:**
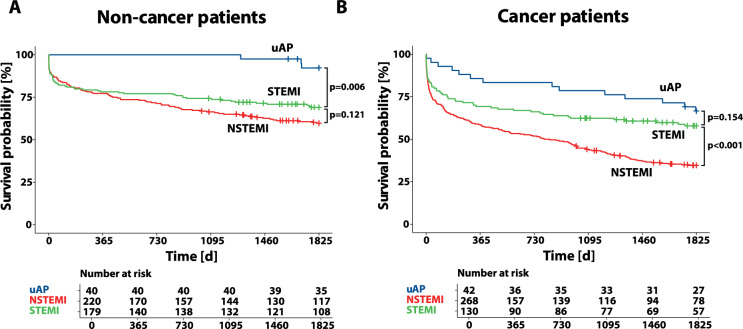
Kaplan Meier survival analysis according to ACS classification (uAP, NSTEMI and STEMI) in (**A**) non-cancer and **B** cancer patients. *P*-values were calculated via the log-rank test

Survival analyses were performed in non-cancer and cancer patients according to ACS classification (uAP, NSTEMI and STEMI). The total 5-year OS was generally lower in cancer patients, whereas uAP patients showed the highest OS and NSTEMI patients the lowest OS in both non-cancer and cancer patients. Differences were particularly pronounced in cancer patients, with mortality rates differing significantly between STEMI and NSTEMI patients primarily in the cancer group (*p* < 0.001, log-rank test). While similar differences between STEMI and NSTEMI mortality were observed in non-cancer patients, they were less pronounced and did not reach statistical significance (*p* = 0.121, log-rank test; Fig. 2). The Kaplan-Meier analysis maintained the same pattern after adjusting for confounders (diabetes, aHT, age > 65 years, male sex, rEF (LVEF ≤ 40%), COPD and stroke (Suppl. Figure 4).

### Cancer is relevant for the survival in ACS patients

Multivariable COX models for prediction of 5-year all-cause mortality including age, ACS type (NSTEMI, STEMI, uAP), kidney dysfunction (GFR < 30 ml/min), anemia (Hb < 10 g/dl), COPD, aHT, diabetes, atrial fibrillation (aFib), reduced ejection fraction (rEF), male sex and prior stroke were built in all patients, including cancer and non-cancer patients.

**Fig. 3:**
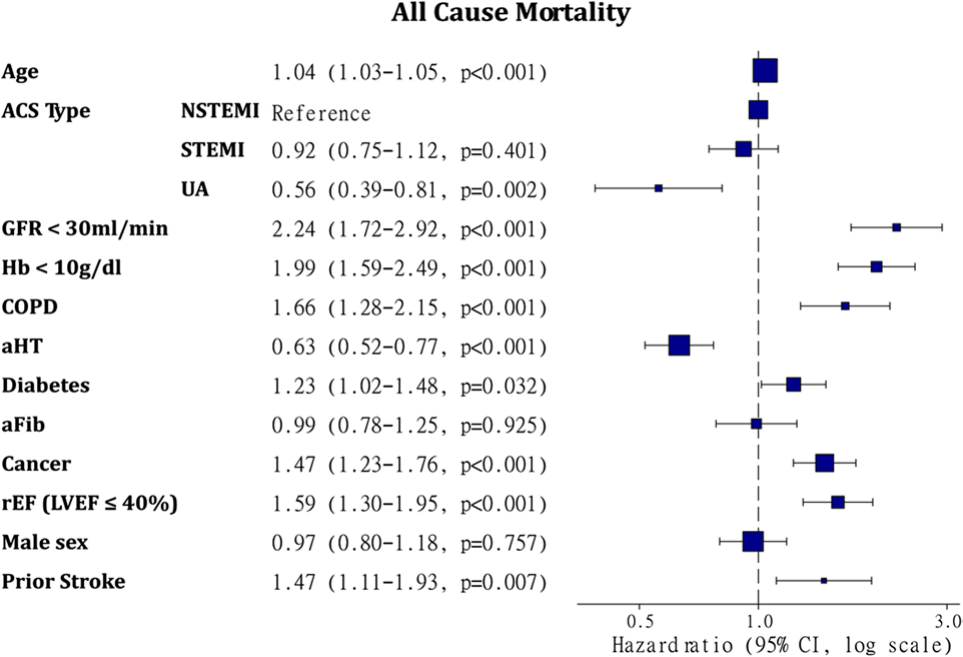
Forest plots representing Cox multivariable logistic regression model for 5-year all-cause mortality in the total ACS group (*n* = 879) including age, ACS type (NSTEMI, STEMI, uAP), GFR < 30 ml/min, Hb < 10 mg/dl, COPD, arterial hypertension (aHT), diabetes, atrial fibrillation (aFib), reduced ejection fraction (rEF), male sex and prior stroke. CI: confidence interval, COPD: chronic obstructive pulmonary disease, GFR: glomerular filtration rate, HR: Hazard ratio, LVEF: left ventricular ejection fraction, rEF: reduced ejection fraction

The diagnosis of cancer was found as an independent predictor (HR: 1.47, CI: 1.23-1.76, *p* < 0.001) of mortality in the ACS patient group. The HR of cancer is comparable to other well-established comorbidities of CAD patients, such as stroke (HR: 1.47, CI: 1.11-1.93, *p* = 0.007) and LVEF ≤ 40% (HR: 1.59, CI: 1.30-1.95, *p* < 0.001). Among the ACS types, NSTEMI was associates with the worst outcome (Fig. 3).

### Predictive value of cardiac comorbidity is preserved in cancer patients with ACS

The same COX model was calculated in the cancer group. We found a preserved predictive value of age (HR: 1.02, CI: 1.01–1.04, *p* = 0.001), GFR < 30 ml/min (HR: 2.14, CI: 1.50–3.01, *p* < 0.001), Hb < 10 g/dl (HR: 2.08, CI: 1.59–2.72, *p* < 0.001), LVEF ≤ 40% (HR: 1.43, CI: 1.09–1.87, *p* = 0.01) and stroke (HR: 2.01, CI: 1.43–2.83, *p* < 0.001).

In the propensity score matched non-cancer group, we found a significant predictive value of age (HR: 1.06, CI: 1.05–1.08, *p* < 0.001), GFR < 30 ml/min (HR: 3.38, CI: 2.12–5.39, *p* < 0.001), COPD (HR: 2.46, CI: 1.62–3.74, *p* < 0.001) and LVEF ≤ 40% (HR: 1.96, CI: 1.43–2.68, *p* < 0.001; Suppl. Figure 5). Apart from prior stroke and anemia, the impact of classical cardiovascular comorbidities, risk factors and cardiac diseases on mortality, reflected in HRs, was higher in the non-cancer group.

### Cardiac readmission is higher in cancer patients

**Fig. 4:**
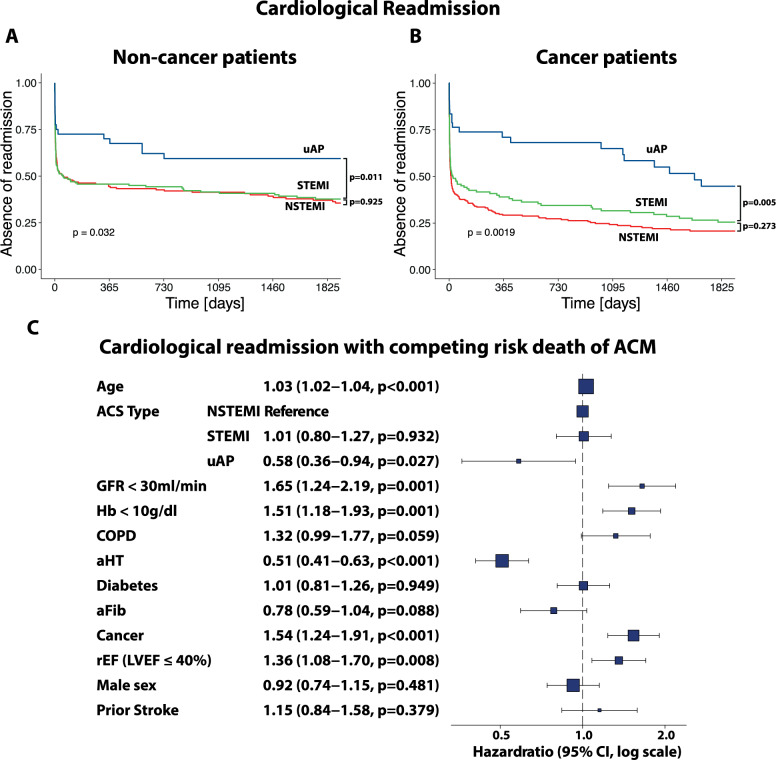
Kaplan Meier plots representing multivariate, competing risk analysis of readmission to a cardiological ward, adjusted to 5-year all-cause mortality (ACM) in (**A**) non-cancer and **B** cancer patients. **C** Forest plot of a multivariate model for cardiological readmission with the competing risk of ACM including age, ACS type (NSTEMI, STEMI, uAP), GFR < 30 ml/min, Hb < 10 mg/dl, COPD, arterial hypertension (aHT), diabetes, atrial fibrillation (aFib), reduced ejection fraction (rEF), male sex and prior stroke. COPD: chronic obstructive pulmonary disease, GFR: glomerular filtration rate, HR: Hazard ratio, LVEF: left ventricular ejection fraction, rEF: reduced ejection fraction

The rate of rehospitalization to a cardiological ward was higher in cancer patients within five years after ACS (71.4% in cancer and 57.6% in non-cancer patients, *p* < 0.001). Compared to STEMI, there was a tendency towards higher readmission rates with NSTEMI in cancer patients (*p* = 0.273, log-rank test) that was not seen in non-cancer patients (*p* = 0.925, log-rank test, Fig. 4A-B). Evaluating the predictive factors influencing cardiac readmission in competing risk analysis of ACM, again we found cancer (HR: 1.54, CI: 1.24–1.91, *p* < 0.001) as an independent determinant, indicating its role in cardiac comorbidity independent of generally higher mortality rates in cancer patients.

Next to cancer, age (HR: 1.03, CI: 1.02–1.04, *p* < 0.001), GFR < 30 ml/min (HR: 1.65, CI: 1.24–2.19, *p* < 0.001), Hb < 10 g/dl (HR: 1.51, CI: 1.18–1.93, *p* = 0.001) and LVEF ≤ 40% (HR: 1.36, CI: 1.08–1.70, *p* = 0.008) were significantly associated with higher readmission rates (Fig. 4C). The same model was calculated in the two groups separately. Kidney dysfunction (HR: 2.47, CI: 1.52–4.01, *p* < 0.001) and rEF (LVEF ≤ 40%) was significantly associated with higher hospitalization only in non-cancer (HR: 1.53: CI: 1.06–2.2, *p* = 0.024). Anemia was only seen in cancer patients with a significant association to readmission (HR: 1.57, CI: 1.18–2.10, *p* = 0.002, Suppl. Figure 6).

### Cardiac biomarkers in risk stratification of ACS patients with cancer

In the clinical routine hs-cTnT was assessed in 528/882 patients and NT-proBNP in 252/882 patients. The clinical characteristics of the patients with available biomarker measurements are shown in Suppl. Tables 1 and 2. Hs-cTnT and NT-proBNP levels are higher in NSTEMI and STEMI patients in line with higher mortality rates compared to uAP patients in the non-cancer and cancer group (Suppl. Figure 7). The predictive value of hs-cTnT and NT-proBNP on 5-year ACM in a subgroup of the patients, where biomarker values were available, was tested. We found a significant association for NT-proBNP with ACM in cancer and non-cancer patients (non-cancer: univariate HR: 1.99, CI: 1.59–2.5, *p* < 0.001; cancer: univariate HR: 1.28, CI: 1.14–1.44, *p* < 0.001), whereas hs-cTnT showed no predictive character in cancer patients with minor effects in the non-cancer group (non-cancer: univariate HR: 1.16, CI: 1.06–1.27, *p* = 0.001, cancer: univariate HR: 1.02, CI: 0.96–1.08, *p* = 0.52). The predictive character of NT-proBNP was preserved when the COX model was adjusted to hs-cTnT (model 1), hs-cTnT and creatinine (model 2), hs-cTnT and rEF (LVEF ≤ 40%) (model 3), hs-cTnT, male sex, age > 65 years, diabetes and aHT (model 4) or hs-cTnT, NSTEMI and STEMI (model 5) in cancer and non-cancer patients (Table 2).

**Table 2:** Univariate and multivariable model evaluating prediction of 5-year all-cause mortality using cardiac biomarker

Non-cancer					Cancer				
Univariate									
Variable	n	Event	HR [CI]	*p*-value	Variable	n	Event	HR [CI]	*p*-value
log2(hs-cTnT)	238	83	1.158 [1.06–1.265]	0.00117	log2(hs-cTnT)	290	158	1.02 [0.961–1.083]	0.521
log2(NT-proBNP)	142	35	1.993 [1.588–2.501]	< 0.0001	log2(NT-proBNP)	110	56	1.28 [1.141–1.435]	< 0.0001
Multivariable									
Model1									
log2(hs-cTnT)	101	26	0.986 [0.842–1.156]	0.866	log2(hs-cTnT)	91	45	0.906 [0.797–1.029]	0.130
log2(NT-proBNP)	101	26	2.73 [1.952–3.818]	< 0.0001	log2(NT-proBNP)	91	45	1.311 [1.15–1.495]	< 0.0001
Model2									
log2(hs-cTnT)	100	25	1.024 [0.857–1.222]	0.7972	log2(hs-cTnT)	77	35	0.917 [0.779–1.078]	0.2939
log2(NT-proBNP)	100	25	2.341 [1.662–3.299]	< 0.0001	log2(NT-proBNP)	77	35	1.197 [1.018–1.409]	0.0298
Creatinine	100	25	1.542 [1.108–2.146]	0.0103	Creatinine	77	35	2.025 [1.064–3.855]	0.0317
Model3									
log2(hs-cTnT)	101	26	1.007 [0.851–1.192]	0.934	log2(hs-cTnT)	91	45	0.903 [0.795–1.026]	0.118582
log2(NT-proBNP)	101	26	2.846 [2.006–4.038]	< 0.0001	log2(NT-proBNP)	91	45	1.288 [1.121–1.478]	0.000336
rEF (LVEF ≤ 40%)	101	26	0.665 [0.286–1.546]	0.343	rEF (LVEF ≤ 40%)	91	45	1.313 [0.696–2.478]	0.400225
Model4									
log2(hs-cTnT)	101	26	1.015 [0.857–1.202]	0.8648	log2(hs-cTnT)	91	45	0.91 [0.794–1.042]	0.170800
log2(NT-proBNP)	101	26	2.762 [1.919–3.975]	< 0.0001	log2(NT-proBNP)	91	45	1.267 [1.1-1.458]	0.000999
Male sex	101	26	1.283 [0.542–3.035]	0.5709	Male sex	91	45	0.909 [0.474–1.746]	0.775485
Age > 65 years	101	26	0.751 [0.082–6.878]	0.8001	Age > 65 years	91	45	2.304 [0.523–10.141]	0.269737
Diabetes	101	26	2.915 [1.226–6.934]	0.0155	Diabetes	91	45	1.123 [0.592–2.131]	0.722043
Hypertension	101	26	0.886 [0.385–2.041]	0.7765	Hypertension	91	45	1.134 [0.57–2.259]	0.719879
Model5									
log2(hs-cTnT)	101	26	0.9 [0.708–1.144]	0.388	log2(hs-cTnT)	91	45	0.939 [0.809–1.089]	0.404
log2(NT-proBNP)	101	26	2.764 [1.985–3.849]	< 0.0001	log2(NT-proBNP)	91	45	1.312 [1.148–1.501]	< 0.0001
NSTEMI	101	26	111467323.131 [0-Inf]	0.997	NSTEMI	91	45	0.74 [0.259–2.114]	0.574
STEMI	101	26	105269425.782 [0-Inf]	0.997	STEMI	91	45	0.51 [0.139–1.866]	0.309

*Hs-cTnT* High-sensitivity cardiac troponin T, *LVEF* Left ventricular ejection fraction, *NSTEMI* Non-ST-elevation myocardial infarction, *NT-proBNP* N-terminal prohormone of brain natriuretic peptide, *STEMI* ST-elevation myocardial infarction

## Discussion

Although the cardiological care of oncological patients has gained more attention in the recent years, the prognostic relevance of ACS in patients with cancer remains insufficiently described.

Our findings indicate that NSTEMI in particular is a risk factor that predisposes for increased ACM rates, especially in cancer patients. We performed propensity score matching according to risk factors, which resulted in comparable groups regarding the coronary status and cardiac function. Nevertheless, cancer patients exhibit a more extensive burden of co-morbidities, such as prior stroke or COPD.

Still, cancer was found to be a relevant, independent predictor for 5-year mortality in all ACS patients that were analyzed. The independent impact of cancer on survival was comparable to other cardiovascular risk factors, such as renal dysfunction, diabetes or heart failure. We observed similar results in terms of readmission due to cardiac events, indicating higher cardiac morbidity in the cancer group. The readmission rate is independent of the generally higher overall mortality observed in cancer patients. The use of cardiac biomarkers for risk stratification was limited in the study patients, but NT-proBNP demonstrated a predictive value in cancer patients.

Little is known about the interaction of ACS and cancer, particularly regarding its impact on long-term survival. Prior studies mostly described in-hospital mortality [22] or short observational periods up to one year [14, 16, 23]. As reported earlier by others, we found as well higher rates of NSTEMI in the cancer group [23]. This may correlate with the higher burden of comorbidities and history of chronic diseases in the cancer group, NSTEMI patients are also known for [24]. 

Frailty, comorbidities, and increased bleeding risks may lead to less invasive treatments in cancer patients and question the impact of ACS on the patients’ outcome. Undertreatment of ACS, especially NSTEMI, has been consistently reported in cancer patients [14, 16, 25]. 

Of note, invasive treatments including PCI (non-cancer: 51.7%, cancer: 51.5%) and CABG (non-cancer: 6.8%, cancer: 4.5%) were comparable in our cancer and non-cancer groups.

In line with data from non-cancer patients, patients with NSTEMI showed the highest mortality [26, 27]. The higher mortality in the NSTEMI group may further be due to less stringent recommendations for immediate invasive diagnostics compared to STEMI and delayed therapies [13]. On the other hand, only approximately 50% of the NSTEMI patients showed a significant coronary stenosis with the need for revascularization.

Interestingly, we found fewer medical treatments, e.g., in terms of antiplatelet treatments, beta-receptor blocker, ACE/AT1-inhibitor and statin use in the cancer group. These drugs are known to be highly relevant for the patients’ mortality [28–30]. Recently, it was shown that the medical therapy could also improve the morbidity and daily life management in terminal cancer patients [31]. The medical undertreatment may be an additional factor next to the higher accumulation of comorbidities that may explain worse outcome of the cancer patients.

Apart from the comorbidity status, we asked if the cardiac biomarkers hs-cTnT and NT-proBNP may predict the outcome in cancer patients with ACS. The two markers have increasing relevance for risk stratification in cancer patients before and during systemic oncological therapies in stable patients [8, 10, 32–34]. Several studies in non-cancer populations have demonstrated a predictive role of NT-proBNP in ACS, particularly in cases of unstable angina (uAP) and NSTEMI [35–38]. 

In the present study, we found NT-proBNP to be predictive for ACM in the non-cancer and cancer group. This finding was maintained by several adjustments to creatinine, hs-cTnT, risk factors, cardiac function and ACS classifications. The predictive role of NT-proBNP in our cancer group may be further explained by the comorbidity burden, including COPD [39]. Hs-cTnT showed a minor predictive value in the present study and only in the non-cancer group. This might be explained by a selection bias of hs-cTnT assessments in only a subset of the patients.

## Conclusions

This study provides the first evaluation of the long-term outcomes in patients with ACS and concomitant cancer. Our findings highlight that ACS, particularly NSTEMI, is associated with reduced survival and increased rehospitalization in cancer patients. Although we could not find evidence for undertreatment in terms of invasive therapies between cancer and non-cancer patients, the high mortality of NSTEMI in general demands precaution in handling these patients.

Despite its retrospective design, the study provides evidence supporting a more thorough evaluation of comorbidities in this patient population, as well as the assessment of cardiac biomarkers, particularly NT-proBNP. Special considerations should be taken, if withholding optimized medical treatments in cancer patients with ACS. Further prospective studies need to warrant these findings.

### Study limitations

There are several limitations of the present study that need to be acknowledged. This is a monocentric, retrospective case control study, which may have led to a selection bias, e.g., the selection of patients in both groups that were subjected to the invasive therapies in the first place. Due to the retrospective nature of the study, there may be also limitations in the homogeneity of the selected data. Cardiac catheterizations of the study patients were performed over a period of eleven years. During this time, there may have been slight differences in the catheterization procedure, even though standardized methods were used at the institution. Some data, such as biomarker measurements (hs-cTnT, NT-proBNP) were not available for every patient. The mean age of the study patients in both, the cancer and non-cancer group, was relatively high in comparison to other clinical studies. This may alter the applicability of the results to other cohorts. There was a lack of data concerning some cardiac risk factors, such as smoking and lipid profiles. We had access to reliable data of ACM and hospitalization, but scarce data on the cause of death, e.g., due to cardiovascular or oncological causes.

## Supplementary Information

Supplementary Material 1.

## Data Availability

No datasets were generated or analysed during the current study.
