# Supplementary material for: The impact of acute coronary syndrome on long-term survival in cancer patients
Source: Cardiooncology. 2026 Feb 7;12:35. doi: 10.1186/s40959-026-00451-9 (PMC12922228; doi:10.1186/s40959-026-00451-9)
Supplement: Supplementary file 1 — Supplementary Material 1. [file 40959_2026_451_MOESM1_ESM.docx]

**Supplemental Information**

**The impact of acute coronary syndrome on long-term survival in cancer patients**

Daniel Finke#^1,2^, Markus B. Heckmann#^1,2^, Jessica M. Schug^1^, Lukas F. Entenmann^1^, Hauke Hund^1^, Hugo A. Katus^1,2^, Norbert Frey^1,2^, Lorenz H. Lehmann^1,2,3*^

*^1^Department of Cardiology, Heidelberg University Hospital, Heidelberg, Germany*

*^2^ German Centre for Cardiovascular Research (DZHK), partner site Heidelberg/Mannheim, Germany*

*^3^ Deutsches Krebsforschungszentrum, Heidelberg (DKFZ)*

# authors contributed equally

**Supplemental Figure 1:**

Propensity score matching of non-cancer patients to cancer patients. Distribution of propensity scores before and after matching in (A) cancer and (B) non-Cancer patients. Number of patients and scores as indicated.

**Suppl. Figure 2:**

(A) Number of patients in the respective LVEF groups separated by cancer status. (B) Number of patients with different degress of coronary artery stenosis separated by cancer status. pEF: preserved ejection fraction, mrEF: mildly reduced ejection fraction, rEF: reduced ejection fraction.

**Suppl. Figure 3:**

Number of NSTEMI patients that received either PCI, ACVB, or no revascularisation. Distributions are shown for all patients (488), as well as only in Non-cancer (220) and in Cancer patients (268). CABG: coronary artery bypass graft. PCI: percutaneous coronary intervention

**Supplemental Figure 4:**

Kaplan Meier survival analysis according to ACS classification (uAP, NSTEMI and STEMI) in cancer and non-cancer patients. Curves are adjusted to diabetes, aHT, CAD, Age > 65, male sex, rEF, COPD and prior stroke using a Cox multivariate model

**Supplemental Figure 5:**

Forest plots representing Cox multivariate logistic regression model for 5-year all-cause mortality in (**A**) non-cancer patients (n=439) and (**B**) cancer patients (n=440) including age, ACS type (NSTEMI, STEMI, uAP), GFR < 30ml/min, Hb < 10 mg/dl, COPD, arterial hypertension (aHT), diabetes, atrial fibrillation (aFib), reduced ejection fraction (rEF), male sex and prior stroke.CI: confidence interval, COPD: chronic obstructive pulmonary disease, GFR: glomerular filtration rate, HR: Hazard ratio, LVEF: left ventricular ejection fraction, NSTEMI: Non-ST elevation myocardial infarction, STEMI: ST-elevation myocardial infarction, uAP: unstable angina pectoris.

**Supplemental Figure 6:**

Forest plots representing readmission to a cardiac ward in 5 years after ACS, with the competing risk of all-cause mortality in (**A**) non-cancer patients and (**B**) cancer patients including age, ACS type (NSTEMI, STEMI, uAP), GFR < 30ml/min, Hb < 10 mg/dl, COPD, arterial hypertension (aHT), diabetes, atrial fibrillation (aFib), reduced ejection fraction (rEF), male sex and prior stroke.CI: confidence interval, COPD: chronic obstructive pulmonary disease, GFR: glomerular filtration rate, HR: Hazard ratio, LVEF: left ventricular ejection fraction, NSTEMI: Non-ST elevation myocardial infarction, STEMI: ST-elevation myocardial infarction, uAP: unstable angina pectoris

**Supplemental Figure 7:**

Box plots representing log2(hs-cTnT) values [ng/l] in (**A**) non-cancer and (**B**) cancer patients and log2(NT-proBNP) values [ng/l] in (**C**) non-cancer and (**D**) cancer patients. T-test was used for statistics: * p ≤ 0.05; ** p≤0.01, **** p≤0.0001.

|  | **Overall**  **(n=528)** | **Non-Cancer**  **(n=238)** | **Cancer**  **(n=290)** | **p-value** |
| --- | --- | --- | --- | --- |
| **Medical history** |  |  |  |  |
| Age (median [IQR]) | 81.00 [72.00, 88.00] | 80.00 [71.00, 87.00] | 81.50 [73.00, 89.00] | 0.111 |
| Male sex (%) | 354 (67.0) | 159 (66.8) | 195 (67.2) | 0.990 |
| Arterial Hypertension (%) | 338 (64.0) | 142 (59.7) | 196 (67.6) | 0.072 |
| Diabetes (%) | 145 (27.5) | 58 (24.4) | 87 (30.0) | 0.179 |
| Atrial fibrillation (%) | 79 (15.0) | 34 (14.3) | 45 (15.6) | 0.788 |
| Heart failure (%) | 61 (11.6) | 23 ( 9.7) | 38 (13.1) | 0.275 |
| COPD (%) | 54 (10.3) | 21 ( 8.9) | 33 (11.4) | 0.414 |
| Stroke (%) | 48 ( 9.1) | 12 ( 5.1) | 36 (12.5) | 0.005 |
| **Cardiac assessment** |  |  |  |  |
| pEF | 99 (18.8) | 48 (20.2) | 51 (17.6) | 0.716 |
| mrEF | 110 (20.8) | 51 (21.4) | 59 (20.3) | 0.796 |
| rEF | 288 (54.5) | 128 (53.8) | 160 (55.2) | 0.518 |
| CAD (any stenosis ≥ 50%) (%) | 449 (85.0) | 206 (86.6) | 243 (83.8) | 0.446 |
| uAP (%) | 40 ( 7.6) | 17 ( 7.1) | 23 ( 7.9) | 0.861 |
| NSTEMI (%) | 280 (53.0) | 110 (46.2) | 170 (58.6) | 0.006 |
| STEMI (%) | 208 (39.4) | 111 (46.6) | 97 (33.4) | 0.003 |
| **Lab. Results (mean±SD)** |  |  |  |  |
| Hb [mg/dl] | 12.50 [10.70, 14.20] | 13.20 [11.53, 14.57] | 12.00 [9.93, 13.60] | <0.001 |
| Leukocytes [number/nl] | 10.23 [8.04, 14.13] | 10.26 [8.10, 13.52] | 10.21 [7.70, 14.56] | 0.892 |
| Platelets [number/nl] | 229.50 [178.00, 283.25] | 229.00 [181.00, 283.00] | 232.00 [176.00, 284.75] | 0.799 |
| **Suppl. Table 1:**  All patients with an available hs-cTnT measurement, divided by cancer status; CAD: coronary artery disease, COPD: chronic obstructive pulmonary disease, mrEF: mildly reduced ejection fraction, NSTEMI: Non-ST-elevation myocardial infarction, pEF: preserved ejection fraction, rEF: reduced ejection fraction, STEMI: ST-elevation myocardial infarction, uAP: unstable Angina. | | | | |

|  | **Overall**  **(n=252)** | **Non-Cancer**  **(n=142)** | **Cancer**  **(n=110)** | **p-value** |
| --- | --- | --- | --- | --- |
| **Medical history** |  |  |  |  |
| Age (median [IQR]) | 79.00 [70.00, 87.00] | 78.50 [69.00, 86.00] | 80.50 [71.50, 88.75] | 0.072 |
| Male sex (%) | 173 (68.7) | 96 (67.6) | 77 (70.0) | 0.788 |
| Arterial Hypertension (%) | 166 (65.9) | 92 (64.8) | 74 (67.3) | 0.781 |
| Diabetes (%) | 75 (29.8) | 40 (28.2) | 35 (31.8) | 0.625 |
| Atrial fibrillation (%) | 46 (18.3) | 26 (18.4) | 20 (18.2) | 1.000 |
| Heart failure (%) | 36 (14.3) | 18 (12.8) | 18 (16.4) | 0.532 |
| COPD (%) | 28 (11.2) | 11 ( 7.8) | 17 (15.5) | 0.087 |
| Stroke (%) | 21 ( 8.4) | 7 ( 5.0) | 14 (12.7) | 0.048 |
| **Cardiac assessment** |  |  |  |  |
| pEF | 65 (25.8) | 39 (27.5) | 26 (23.6) | 0.313 |
| mrEF | 57 (22.6) | 31 (21.8) | 26 (23.6) | 0.956 |
| rEF | 112 (44.4) | 58 (40.8) | 54 (49.1) | 0.391 |
| CAD (any stenosis ≥ 50%) (%) | 211 (83.7) | 118 (83.1) | 93 (84.5) | 0.891 |
| uAP (%) | 30 (11.9) | 16 (11.3) | 14 (12.7) | 0.874 |
| NSTEMI (%) | 138 (54.8) | 67 (47.2) | 71 (64.5) | 0.009 |
| STEMI (%) | 84 (33.3) | 59 (41.5) | 25 (22.7) | 0.003 |
| **Lab. Results (mean±SD)** |  |  |  |  |
| Hb [mg/dl] | 12.90 [11.00, 14.50] | 13.65 [11.80, 14.70] | 12.10 [9.77, 13.88] | <0.001 |
| Leukocytes [number/nl] | 9.68 [7.68, 12.91] | 9.90 [8.07, 12.96] | 8.68 [7.23, 12.76] | 0.056 |
| Platelets [number/nl] | 228.50 [177.75, 277.25] | 229.00 [187.75, 276.50] | 224.00 [162.50, 277.25] | 0.398 |
| **Suppl. Table 2:**  All patients with an available NT-proBNP measurement, divided by cancer status; CAD: coronary artery disease, COPD: chronic obstructive pulmonary disease, mrEF: mildly reduced ejection fraction, NSTEMI: Non-ST-elevation myocardial infarction, pEF: preserved ejection fraction, rEF: reduced ejection fraction, STEMI: ST-elevation myocardial infarction, uAP: unstable Angina. | | | | |
